# Acknowledgement to Reviewers 2024

**DOI:** 10.3389/phrs.2025.1608758

**Published:** 2025-07-11

**Authors:** PHR Editorial Office

**Affiliations:** Swiss School of Public Health (SSPH+), Zürich, Switzerland

The Editors of *Public Health Reviews* would like to thank all Reviewers in 2024 for the time they have spent and the valuable contributions they have made to ensure the scientific quality of the journal.

Muhammad Hasan Abid, United States

Ojo Agunbiade, Nigeria

Divine Ahadzie, Ghana

Luciene Almeida, Brazil

Diogo Almeida, Portugal

Saeed Anwar, Canada

Natália Araújo, Portugal

John Koku Awoonor-Williams, Ghana

Ana Azevedo, Portugal

Suzanne Babich, United States

Helen Banks, Italy

Mrittika Barua, Bangladesh

Stefanie Beck, Germany

Nicole Bizzotto, Switzerland

Dina Boulos, Egypt

Stefan Brockmann, Germany

Aurora Bueno-Cavanillas, Spain

Diana Buitrago-Garcia, Switzerland

Maria Bujnowska-Fedak, Poland

Maria Vittoria Bulgheroni, Italy

Chepkoech Buttia, Switzerland

Tess Carter, United States

Laura Ines Plata Casas, Colombia

Arohi Chauhan, India

José Chen-Xu, Spain

Emma Clarke-Deelder, Switzerland

Rinat Cohen, Israel

Virgínia Conceição, Portugal

Ana Cruz, Portugal

Andrew Dannenberg, United States

Mark Dayer, Ireland

Jason Domogauer, United States

Xuejie Dong, China

Amol Dongre, India

Didier Ekouevi, France

Ana Escoval, Portugal

Marta Fadda, Switzerland

Angela Freitas, Portugal

Desmond Gagakuma, Ghana

Pedro Gallo, Spain

Viktoria Gastens, Switzerland

Kritika Gupta, United States

Ana Gutierrez, Switzerland

Ben Harris-Roxas, Australia

Herwansyah Herwansyah, Netherlands

Songhua Hu, United States

Kazuhiko Ito, United States

Alex Ayebazibwe Kakama, Uganda

Ariel Kalil, United States

Gaige Kerr, United States

Roxanne Keynejad, United Kingdom

Zahra Khorrami, Iran

Valentina Kieseppä, Finland

Nino Kuenzli, Switzerland

Barry Levy, United States

Hooi Min Lim, Malaysia

Theo Lorenc, United Kingdom

Raquel Lucas, Portugal

António Machado, Portugal

Laura Magaña, United States

Muhammad Mahadzir, Singapore

Olusesan Makinde, South Africa

Svenn-Erik Mamelund, Norway

Nicole Mauer, Belgium

Steven Meanley, United States

Niklaus Meier, Switzerland

Tadesse Misgana, Ethiopia

Mahdiyeh Mohammadzadeh, Iran

Samantha Morais, Portugal

Rafaella Moreira, Brazil

Sapna Negi, India

Ahmed Newera, Saudi Arabia

Patience Nyakato, South Africa

Osondu Ogbuoji, United States

Adeyemi Okunogbe, United States

George Otieno, Kenya

Thomas O'Toole, United Kingdom

Samuel Asiedu Owusu, Ghana

Inês Paciência, Finland

Takis Panagiotopoulos, Greece

Pierre-Marie Preux, France

Ratna Chrismiari Purwestri, Czechia

Cristina Hernandez Quevedo, United Kingdom

Amber Raja, United Kingdom

Rama Shankar Rath, India

Pedro Reis, Brazil

Priscilla Robinson, Australia

Miguel Rocha, Portugal

Pablo Rodríguez-Feria, Netherlands

Anna Rosofsky, United States

João Cavaleiro Rufo, Portugal

Ignacio De Loyola González Salgado, Spain

Aliya Sarmanova, Switzerland

Angela Schnelli, Switzerland

Patricia Schwärzler, Switzerland

Reihane Sharif, Iran

Pratik Shingru, United States

Tuulikki Sjögren, Finland

Juley De Smidt, South Africa

Sara Soares, Portugal

Camila Squarcini, Brazil

Cristina Teixeira, Portugal

Raquel Teixeira, Portugal

Luis Cereijo Tejedor, Spain

Roba Argaw Tessema, Ethiopia

Lyle Turner, Australia

Carol Underwood, United States

Roberto Valiente, United Kingdom

Costas A. Varotsos, Greece

Helena Velez-Botero, Colombia

Vicent Villanueva, Spain

Edgar Whitley, United Kingdom

Frank Wieber, Switzerland

Sharon Wolf, United States

Jiawei Zhang, Denmark

Simeon Joel Zürcher, Switzerland

